# Supplementary material for: Global analysis of ocean phytoplankton nutrient limitation reveals high prevalence of co-limitation
Source: Nat Commun. 2023 Aug 17;14:5014. doi: 10.1038/s41467-023-40774-0 (PMC10435517; doi:10.1038/s41467-023-40774-0)
Supplement: Supplementary file 1 — Supplementary Information [file 41467_2023_40774_MOESM1_ESM.pdf]

Supplementary Text 1

Correlation statistics using the maximum observed chlorophyll-a based growth rate (i.e., the growth rate for treatment with strongest positive response) across all experiments as the response variable showed significant correlations with chlorophyll-a (Chl), log_{10}(Chl), Temperature, and Latitude (Table S1). Correlations with Chl, log_{10}(Chl) were furthermore stronger for data where at least 2 nutrients were added as response variable (i.e., where the potential for remaining nutrient limitation to be suppressing the observed maximum growth rate is reduced) (Table S2).

Table S1. Correlation statistics using the maximum observed chlorophyll-a based growth rate (i.e., the growth rate for treatment with strongest positive response) across all experiments as response variable.

	**Spearman r**	**Pearson r**	**p**	**n**
**Chl**	-0.405	-0.285	0.0036	103
**Log_{10}(Chl)**	-0.405	-0.621	2.6×10^{-12}	103
**Temperature**	0.445	0.506	5.0×10^{-8}	103
**Latitude**	-0.308	-0.411	1.6×10^{-5}	103

Table S2. Correlation statistics using maximum observed chlorophyll-a based growth rate in all experiments where at least 2 nutrients were added as response variable (i.e., where the potential for remaining nutrient limitation to be supressing the observed maximum growth rate is reduced).

	**Spearman r**	**Pearson r**	**p**	**n**
**Chl**	-0.472	-0.292	0.0136	71
**Log_{10}(Chl)**	-0.472	-0.650	8.6×10^{-10}	71
**Temperature**	0.413	0.445	1.0×10^{-4}	71
**Latitude**	-0.199	-0.320	0.0065	71

Stepwise linear regression with log_{10}(Chl), Temperature and Latitude as predictors only returned log_{10}(Chl) as a significant predictor (i.e., no more variance was explained at $p<0.05$ by adding in temperature or latitude. Stepwise linear regression with Chl, Temperature and Latitude as predictors only returned Temperature as significant predictor (i.e., no more variance explained at $p<0.05$ by adding in Chl or latitude).

When the temperature normalised maximum growth rate observed in each experiment was used as the response variable (see Methods), neither Chl (or log_{10}(Chl)) or Latitude were significantly correlated (i.e., residuals following temperature normalisation were not correlated with Chl or log_{10}(Chl)).
