# Supplementary material for: Global analysis of ocean phytoplankton nutrient limitation reveals high prevalence of co-limitation
Source: Nat Commun. 2023 Aug 17;14:5014. doi: 10.1038/s41467-023-40774-0 (PMC10435517; doi:10.1038/s41467-023-40774-0)
Supplement: Supplementary file 3 — Description of additional supplementary files [file 41467_2023_40774_MOESM3_ESM.pdf]

<u>Description of additional supplementary files</u>

Supplementary Data 1: The compiled experimental dataset.
