# Supplementary material for: Global analysis of ocean phytoplankton nutrient limitation reveals high prevalence of co-limitation
Source: Nat Commun. 2023 Aug 17;14:5014. doi: 10.1038/s41467-023-40774-0 (PMC10435517; doi:10.1038/s41467-023-40774-0)
Supplement: Supplementary file 5 — Reporting Summary [file 41467_2023_40774_MOESM5_ESM.pdf]

nature portfolio

Corresponding author(s): Thomas Browning
Mark Moore

Last updated by author(s): Jul 20, 2023

Reporting Summary

Nature Portfolio wishes to improve the reproducibility of the work that we publish. This form provides structure for consistency and transparency in reporting. For further information on Nature Portfolio policies, see our Editorial Policies and the Editorial Policy Checklist.

Statistics

For all statistical analyses, confirm that the following items are present in the figure legend, table legend, main text, or Methods section.

n/a	Confirmed	
☐	☒	The exact sample size (*n*) for each experimental group/condition, given as a discrete number and unit of measurement
☐	☒	A statement on whether measurements were taken from distinct samples or whether the same sample was measured repeatedly
☐	☒	The statistical test(s) used AND whether they are one- or two-sided<br>*Only common tests should be described solely by name; describe more complex techniques in the Methods section.*
☒	☐	A description of all covariates tested
☒	☐	A description of any assumptions or corrections, such as tests of normality and adjustment for multiple comparisons
☐	☒	A full description of the statistical parameters including central tendency (e.g. means) or other basic estimates (e.g. regression coefficient) AND variation (e.g. standard deviation) or associated estimates of uncertainty (e.g. confidence intervals)
☐	☒	For null hypothesis testing, the test statistic (e.g. *F*, *t*, *r*) with confidence intervals, effect sizes, degrees of freedom and *P* value noted<br>*Give P values as exact values whenever suitable.*
☒	☐	For Bayesian analysis, information on the choice of priors and Markov chain Monte Carlo settings
☒	☐	For hierarchical and complex designs, identification of the appropriate level for tests and full reporting of outcomes
☒	☐	Estimates of effect sizes (e.g. Cohen's *d*, Pearson's *r*), indicating how they were calculated

Our web collection on statistics for biologists contains articles on many of the points above.

Software and code

Policy information about availability of computer code

Data collection	No software used
Data analysis	Some data were extracted from plots in publications using a web-based extractor (https://automeris.io/WebPlotDigitizer). Statistics, other calculations, and plotting were conducted using R version 4.1.0 (using packages 'base', 'stats', 'maps', 'mapdata', 'raster' and 'fields').

For manuscripts utilizing custom algorithms or software that are central to the research but not yet described in published literature, software must be made available to editors and reviewers. We strongly encourage code deposition in a community repository (e.g. GitHub). See the Nature Portfolio guidelines for submitting code & software for further information.

Data

Policy information about availability of data

All manuscripts must include a data availability statement. This statement should provide the following information, where applicable:

- Accession codes, unique identifiers, or web links for publicly available datasets
- A description of any restrictions on data availability
- For clinical datasets or third party data, please ensure that the statement adheres to our policy

The bioassay experiment dataset generated in this study (Supplementary Data 1) has been deposited in the Zenodo database under accession code: https://doi.org/10.5281/zenodo.7937742. The GEOTRACES Intermediate Data Product is available from the British Oceanographic Data Centre (BODC): https://www.bodc.ac.uk/geotraces/data/idp2017/.

Human research participants

Policy information about studies involving human research participants and Sex and Gender in Research.

Reporting on sex and gender	NA
Population characteristics	NA
Recruitment	NA
Ethics oversight	NA

Note that full information on the approval of the study protocol must also be provided in the manuscript.

Field-specific reporting

Please select the one below that is the best fit for your research. If you are not sure, read the appropriate sections before making your selection.

☐ Life sciences ☐ Behavioural & social sciences ☒ Ecological, evolutionary & environmental sciences

For a reference copy of the document with all sections, see nature.com/documents/nr-reporting-summary-flat.pdf

Ecological, evolutionary & environmental sciences study design

All studies must disclose on these points even when the disclosure is negative.

Study description	The results of oceanic nutrient enrichment bioassay experiments were collated from the literature alongside associated meta data (where available). This dataset was then analyzed to globally assess spatial patterns of oceanic nutrient limitation, quantitatively evaluate the response of phytoplankton to nutrient supply, and assess potential drivers. Only studies where nutrient enrichments were conducted with at least triplicate independent biological replicates were included in the dataset, apart from a small number of mesoscale (km scale) oceanic iron enrichment experiments that were also included. The assembled dataset included experiments conducted with both factorial and non-factorial sets of nutrient additions.
Research sample	Experimental data was synthesized from the literature. Only studies where nutrient enrichments were conducted with at least triplicate replication were included in the dataset, apart from a small number of mesoscale (km scale) oceanic iron enrichment experiments that were also included. The complete dataset, with accompanying references for each individual experimental treatment, is provided in both Supplementary Data 1 and uploaded to Zenodo (see 'Data availability' statement).
Sampling strategy	All experiments found in the literature conforming to the requirements described above were included.
Data collection	Data were either extracted from the manuscript itself, obtained directly from the authors of the relevant study, a web-based data extractor tool was used to obtain data from publication figures (https://automeris.io/WebPlotDigitizer), or these data were not included in the dataset. Data collation was conducted by T. Browning, M. Moore, and G. Vickery.
Timing and spatial scale	Data collation (that is, assembly of the bioassay experiment dataset) was conducted between 2019 and 2021 with irregular frequency (determined by time available for this project and as new studies became available in the literature).
Data exclusions	No data that fit the requirements outlined below (for treatment replication) were excluded from the analysis.
Reproducibility	Only experiments in the literature using at least triplicate replication were included in the dataset, apart from a small number of mesoscale (km scale) oceanic iron enrichment experiments that were also included. Attempts at replication by the authors of these studies were assumed successful in all cases.
Randomization	No randomization was used in the data analysis.
Blinding	The investigators were not blinded during dataset assembly (that is, extraction of the bioassay data from the various publications), however, further data analysis with the assembled dataset was conducted without information about either the authors or manuscript titles of the individual source studies.

Did the study involve field work? ☐ Yes ☒ No

Reporting for specific materials, systems and methods

We require information from authors about some types of materials, experimental systems and methods used in many studies. Here, indicate whether each material, system or method listed is relevant to your study. If you are not sure if a list item applies to your research, read the appropriate section before selecting a response.

Materials & experimental systems

n/a	Involved in the study
☒	☐ Antibodies
☒	☐ Eukaryotic cell lines
☒	☐ Palaeontology and archaeology
☒	☐ Animals and other organisms
☒	☐ Clinical data
☒	☐ Dual use research of concern

Methods

n/a	Involved in the study
☒	☐ ChIP-seq
☒	☐ Flow cytometry
☒	☐ MRI-based neuroimaging
